# Phylogeny and divergence time estimation of the subfamily Amphipsyllinae based on the *Frontopsylla diqingensis* mitogenome

**DOI:** 10.3389/fvets.2024.1494204

**Published:** 2024-12-11

**Authors:** Ju Pu, Xiaoxia Lin, Wenge Dong

**Affiliations:** Yunnan Provincial Key Laboratory for Zoonosis Control and Prevention, Institute of Pathogens and Vectors, Dali University, Dali, China

**Keywords:** Amphipsyllinae, mitogenome, plague, phylogeny, divergence time

## Abstract

Fleas are primarily parasites of small mammals and serve as essential vectors of the transmission of plague. The subfamily Amphipsyllinae (Siphonaptera: Leptopsyllidae) consists of 182 species across 13 genera, widely distributed worldwide. Only two species of Amphipsyllinae have been sequenced for complete mitogenomes to date. It hinders the taxonomy and evolutionary history studies of fleas. In this study, we first sequenced the *Frontopsylla diqingensis* mitogenome and performed comparative mitogenomic analyses with the two other species (*Frontopsylla spadix* and *Paradoxopsyllus custodis*) in Amphipsyllinae available in the NCBI database. The evolutionary process of Amphipsyllinae was comprehensively analyzed in terms of nucleotide composition, codon usage, nucleotide diversity, tRNA secondary structure, nucleotide skew, phylogeny tree, and divergence time. Nucleotide diversity and tRNAs of three species of fleas of Amphipsyllinae have differences among different species. The effective number of codon (ENC)-plot, neutrality curve, PR2, and correspondence analysis (COA) showed that the codon preference of Amphipsyllinae was influenced mainly by natural selection. For phylogenetic trees and divergence time of the order Siphonaptera, our results showed two concatenated data matrices, namely, PCG: (((Ceratophyllidae + Leptopsyllidae) + ((Vermipsyllidae + Hystrichopsyllidae) + Ctenophthalmidae)) + (Pulicidae + Pygiopsyllidae)); PCGRNA: ((((Ceratophyllidae + Leptopsyllidae) + ((Vermipsyllidae + Hystrichopsyllidae) + Ctenophthalmidae)) + Pulicidae) + Pygiopsyllidae). We concluded that *P. custodis* and *Macrostylophora euteles* from GenBank are the same species by phylogenetic trees and sequence alignment, and supported the monophyly of Amphipsyllinae. Amphipsyllinae diverged in the Cenozoic, approximately 73.37–40.32 million years ago (Mya). The majority of the species within the intraordinal divergence into extant lineages occurred after the K-Pg boundary. The common ancestor of the extant order Siphonaptera diverged during the Cretaceous. Our findings supported those of Zhu et al. (1). This study provides new insights into the evolutionary history and taxonomy of the order Siphonaptera.

## 1 Introduction

The order Siphonaptera, commonly known as fleas, feed on host blood and serve as vectors of plague, making them significant medical insects worldwide. Recent molecular studies have demonstrated they are a monophyletic group (1). The origin of Siphonaptera can be traced back to the Mesozoic Jurassic (2). However, Zhu et al. (1) suggested that the common ancestor of Siphonaptera began to diverge in the Cretaceous. Due to the scarcity of fossil records, the early evolution of fleas is rarely known, and the earliest fossils of fleas recorded and identified were in the Eocene and Miocene (*Palaeopsylla klebsiana*, *Palaeopsylla dissimilis*, *Peusianapsylla baltica*, and *Peusianapsylla groehni* from Baltic amber) (3–8). Fleas are species-rich and complex in morphology, and their origin and taxonomic status have been controversial. In the 19th century, some researchers classified fleas as beetles based on their morphological characteristics (9). In the 20th century, some researchers suggested that fleas were closely related to the Orders Mecoptera and Diptera, constituting the group Antliophora (10). In the 21st century, it was found that the orders Siphonaptera and Mecoptera were sister groups (11, 12), but some researchers supported that the order Siphonaptera and the family Nannochoristidea (Mecoptera) were sister groups (13). Wu et al. (14) presumed that some genera and species of the families Macropsyllidae, Stephanocircidae, and Pygiopsyllidae (currently a subfamily of Hystrichopsyllidae) at the base of tree parasitized the older host (Basidioidea and Monoporida), while the family Ceratophyllidae at the top of tree parasitized the younger host (Lagomorpha, Rodentia, Eulipotyphla, etc.,). Such an alignment differs from that studied by Hopkins and Rothschild (14). Currently, more than 2,500 species of Siphonaptera are regarded as valid species or subspecies—Lewis et al. (15) conjectured that the genera and species described are approximately 250–300 species. The drastic changes in the natural environment and climate in recent years have resulted in some host species being critically endangered or even extinct. Some flea species with high host specificity, such as those in Ceratophyllidae, Ischnopsyllidae, Stephanocircidae, and Pygiopsyllidae families, may also be at risk of extinction. Fleas are temporary hosts or important vectors for many zoonotic diseases, such as *Yersinia pesti*, *Rickettsia typhi*, and *Bartonella henselae* (16–18). The evolutionary history, taxonomic status, and transmission of plague of fleas are worth studying.

The head of the subfamily Amphipsyllinae (Siphonaptera: Leptopsyllidae) is integricipit, with an antennal fossa that may be proximal to the top of the head and lacks cleft. Usually, there is no genal comb; if there is a genal comb, there is only one or two spines backward. Amphipsyllinae with 5 tribes, 13 genera, and 182 species (subspecies) distributed mainly in Palaearctic (mostly) and Neopelagic (14). However, (15, 19) classified Amphipsyllinae into 23 genera and 177 species, with the vast majority parasitizing mammals, and only a few parasitizing birds. Previously, the subfamily Leptopsyllinae, characterized by a genal comb, was often included in the family Hystrichopsyllidae, while Amphipsyllinae lacking a genal comb was included in Ceratophyllidae. It was later found that Amphipsyllinae and Leptopsyllinae were more closely related, and the two subfamilies lumped into one family (Leptopsyllidae). So far, the complete mitogenomes have been determined for only three Amphipsyllinae species. Mitogenomes with simple structure, maternal inheritance, and fast evolution were used to study the phylogeny, molecular evolution, insect taxonomy, and population genetics of insects (20–23). The typical insect mitogenome contains 37 genes (13 protein-coding genes, 22 tRNA genes, and 2 rRNA genes) and one non-coding region. Thirteen of these protein-coding genes are respiratory chain components in the inner mitochondrial membrane. Transfer tRNA (tRNAs) usually possess typical cloverleaf structures, consisting of an amino acid acceptor arm, anticodon arm, dihydrouracil arm (DHU), and TψC arm. Typically, the amino acid acceptor arm is 7 bp, the DHU arm is 4 bp, TψC arm is 5 bp, and the anticodon arm is 5 bp. rRNAs (*rrnL* and *rrnS*) are primarily used in phylogenetic studies (24). Non-coding regions, also called control regions, are the regulatory regions for replication and transcription initiation of mitogenomes and have the fastest rate of evolution (25, 26).

In this study, (a) the mitogenome of *Frontopsylla diqingensis* Li and Hsieh, 1974, was determined and analyzed for the first time, with its morphological characteristics described in detail; (b) in combination with the mitogenome sequences of Amphipsyllinae species available in GenBank (*Frontopsylla spadix* Jordan and Rothschild, 1921: NC073018 and *Paradoxopsyllus custodis* Jordan, 1932: OQ627398), we analyzed the structural features and variation of the mitogenomes of Amphipsyllinae to further clarify the evolutionary differences among them; and (c) the phylogenetic relationships and origin of Siphonaptera were evaluated, and the divergence times among various groups of Siphonaptera were estimated.

## 2 Materials and methods

### 2.1 Specimen collection, morphological identification, DNA extraction, and mitogenome sequencing

In May 2012, *Ochotona thibetana* Milne and Edwards, 1871, (Lagomorpha, Ochotonidae) was captured with mouse cages in Diqing Tibetan Autonomous Prefecture, Yunnan Province, China, and taken to the laboratory for ectoparasite collection. All specimens of *F. diqingensis* were collected on *O. thibetana* and stored in an Eppendorf tube with 95% ethanol at −80°C for subsequent identification and DNA extraction. *F. diqingensis* and *O. thibetana* were deposited at Dali University. All specimens were trapped and preserved with the approval of the Animal Ethics Committee of Dali University under the approval number MECDU-201912-20.

The morphological identification is primarily based on “*Fauna Sinica insecta Siphonaptera*” (14). For specimens to be photographed, we first placed them in distilled water to clean them and to stretch the wrinkled fleas. The specimens were sealed and dried, then photographed and identified in detail for morphological features using an OLYMPUS (SZ2-ILST) micrographic system. Five specimens were sent to Shanghai Winnerbio Technology Co., Ltd. (Shanghai, China). The tissues were cryoprocessed, ground, and mixed with cetyltrimethylammonium bromide (CTAB). CTAB is a cationic active agent that lysed membrane proteins and lipids, as well as depolymerized nuclear proteins. CTAB-nucleic acid complexes are soluble and stable at high salt (>0.7 mM) concentrations. Still, at low salt concentrations (0.1–0.5-mM NaCl), CTAB-nucleic acid complexes precipitate due to reduced solubility, while the majority of the proteins, polysaccharides, etc. remain dissolved in the solution. The CTAB-nucleic acid complex is separated from sugars, proteins, etc., by centrifugation, and nucleic acids can be precipitated by ethanol, while CTAB dissolved in ethanol and removed. DNA concentration and purity were measured using a spectrophotometer and electrophoretically tested for completeness. Sequencing was performed on the Illumina NovoSeq 6,000 platform (Illumina, Inc.). During Illumina sequencing, a sequencing library was constructed using about 1 μg of genomic DNA. According to the manufacturing protocol, the DNA samples were sheared into 400–500-bp fragments using a Covaris M220 Focused Acoustic Shear (Covaris, Inc.). The sheared fragments were used to prepare Illumina sequencing libraries. The prepared library was then used for paired-end Illumina sequencing (2 × 150 bp) on an Illumina NovaSeq 6,000 platform.

### 2.2 Mitochondrial sequence assembly, annotation, and analysis of *F. diqingensis*

MitoZ 2.3 (27) was used to assemble the mitogenome, and BWA v0.7.17 (28) and Samtools v0.1.20 (29) were used to assess the confidence of the assembly data (sequencing depth ≥ 100×). Annotation was performed using Geneious Prime 11.0 (30). Protein-coding genes and rRNA genes were identified by BLAST2.16 (31) and MITOS Web Server (32). The tRNA gene was predicted using tRNAscan SE2.0 (33) and ARWEN (34), and then manually checked and corrected. The annotated mitogenome sequence of *F. diqingensis* has been deposited to GenBank (accession number: PP083946).

CodonW was used to analyze the codon preference of three species. Skew was calculated as AT skew = (A − T)/(A + T) and GC skew = (G − C)/(G + C). ClustalX (35) and MEGA (36) were used to calculate the conserved and variant sites of tRNA genes; DnaSP v5 (37) was used to analyze the nucleotide diversity, variable site, conserved site, and singleton site of 13 protein-coding genes.

### 2.3 Phylogenetic analysis

*Boreus elegans* Carpenter, 1935 (38) was used as the outgroup. Bayesian inference (BI) (39) tree and Maximum likelihood (ML) (40) tree were constructed based on two concatenated data matrices (PCG: 13 protein-coding genes; PCGRNA: 13 protein-coding genes and 2 rRNA gene sequences) of 23 species (Supplementary Table S1). Sequence alignment was initially performed using MAFFT (41) software and then optimized using MACSE v 2.06 (42) software. MACSE uses the classic “Needleman–Wunsch” algorithm to correctly identify pseudogenization events and maintain the ancestral codon structure. Since comparing PCG sequences directly using MACSE was too slow, the PCG sequences were first compared using MAFFT and then optimized using MACSE. Significant gaps and ambiguous sites were omitted using Gblocks (43). The best-fit nucleotide substitution model for the tree was determined using ModelFinder (44). The best-fit nucleotide substitution model for constructing ML and BI trees is shown in Supplementary Table S2. IQ-TREE (45) was used to construct the ML tree. The number of bootstrap repetitions in the maximum likelihood analysis was set to 1,000, and the support rate of the node branch uses the maximum likelihood bootstrap proportion (BSP). When BSP ≥ 70%, the node has a high support rate. MrBayes 3.2.6 (39) was used to construct the BI tree. The BI tree underwent 3,000,000 generations, with trees sampled every 1,000, each with four independent Markov Chain Monte Carlo (MCMC). When Bayesian posterior probabilities (BPP) ≥ 95%, the posterior probability had a higher support rate. Figtree (46) was used to visualize and edit the evolutionary tree.

### 2.4 Divergence time estimation

Based on the reconstructed phylogenetic tree, the divergence time of 23 species of 15 genera in 7 flea families was estimated using Bayesian Evolutionary Analysis Sampling Trees (BEAST 2.0) (47), with data from 13 PCGs. Due to limited flea fossil records and available sequence data, fossil calibrations (amber specimens, see Introduction section) were employed to confirm true Siphonaptera, which convey verifiable knowledge of the existence of specific lineages of Siphonaptera over time. (1) The genus *Pulex* Linnaeus, 1758. Although this flea fossil was placed into the Miocene, the age range of Dominican amber was controversial, with estimates running from 20 to 15 Mya (Iturralde–Vinent and MacPhee, 1996) to 45–30 Mya (Schlee, 1990). It was set to 35–15 Mya to reflect this uncertainty (1, 48). (2) Ctenophthalmidae: 40–35 Mya was used for calibration points, and the root node was not calibrated. First, the best model for accurate phylogenetic estimation was selected using ModeFinder (44). The most suitable replacement model, GTR, was set in BEAUti, along with the adoption of a strict molecular clock model. The tree prior model used the Yule process, and the prior distribution was set to normal to estimate the divergence time. The Markov chain (MCMC chain) ran for 40,000,000 generations, with trees sampled every 1,000 generations. The convergence of the parameter estimates was performed using Tracer. The ESS value of the parameters was >200. The top 10% of the tree was discarded as burn-in. Trees were edited and visualized using tvBOT (49).

## 3 Results

### 3.1 Morphological characteristics and mitogenome organization of *F. diqingensis*

**Figure 1:**
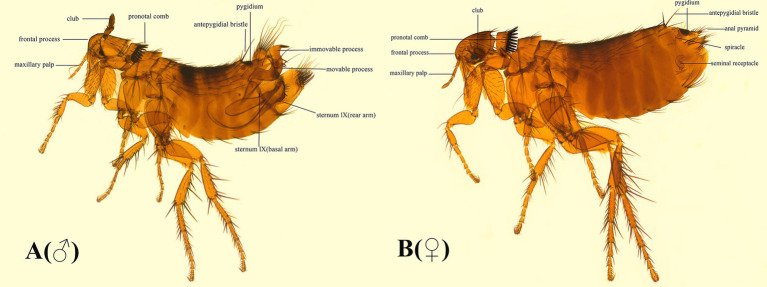
Morphological characteristics of **(A)** (♂) and **(B)** (♀) of *Frontopsylla diqingensis* from China.

From a careful examination of specimens of *F. diqingensis*, the following characteristics were used for identification: The sternum IX (rear arm) is not developed and narrow. It bears six bristles. There is a row of seven bristles above it, and the spacing between the two bristles is very short. The top of the immovable process is up to the midpoint of the movable process, which is higher than *F. spadix* Jordan and Rothschild, 1921. The immovable process is conical, and its upper anterior angle is mostly a right angle. The concave degree of the upper part of the trailing edge of the movable process is usually deeper. There are three sub-thorns in the middle and lower part of the trailing edge of the movable process. There are three spiniforms in the middle and lower part of the trailing edge of the movable protrusion. The shape of slermum VII (rear arm) differs from *F. spadix*. The tail boundary of spermatheca is unclear (Figure 1).

**Figure 2:**
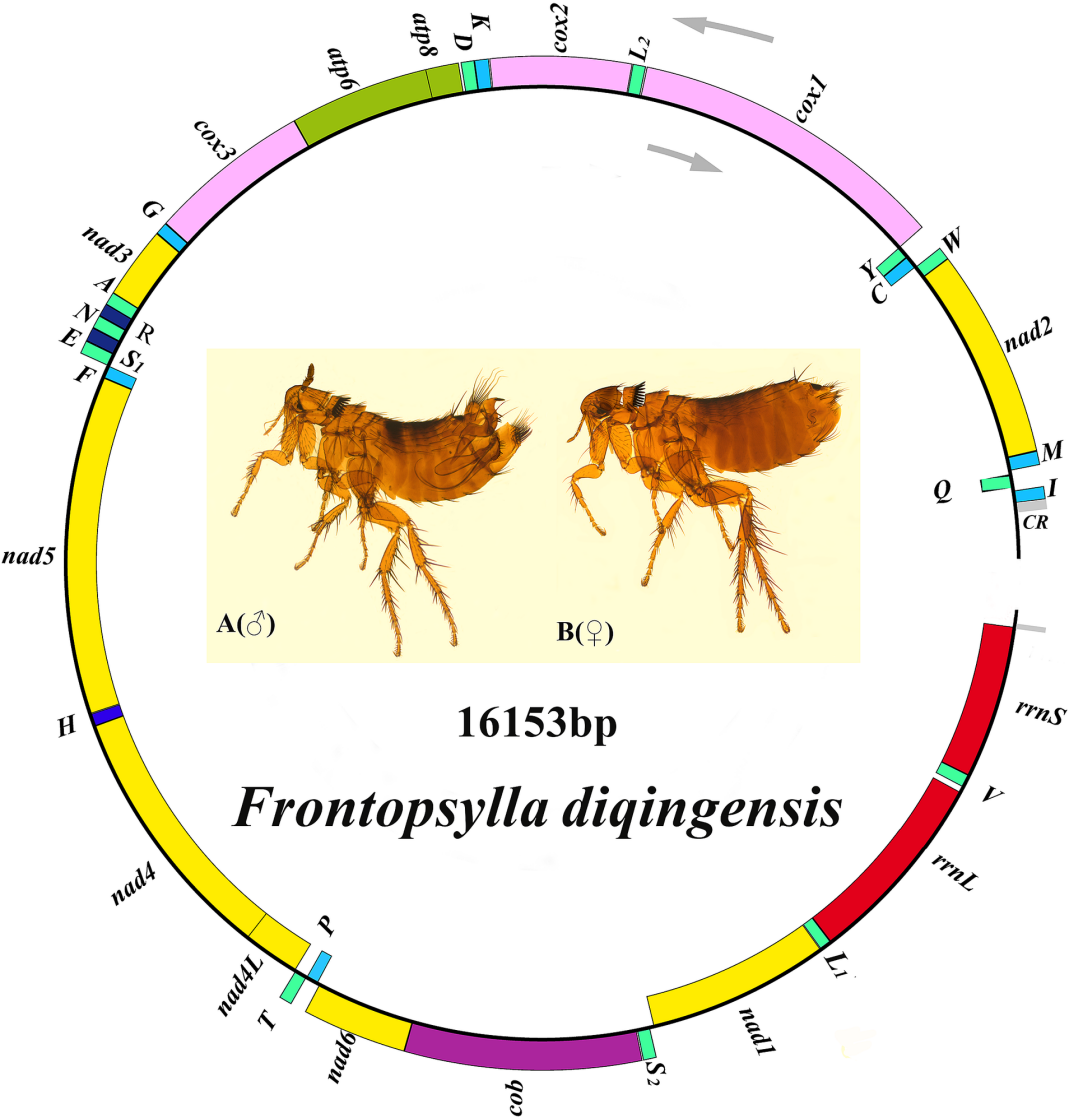
Organization of the *Frontopsylla diqingensis* mitogenome. tRNA genes were shown with the single-letter abbreviations of their corresponding amino acids. The two leucines are denoted by L_1_ (tag) and L_2_ (taa), and the two serines are denoted by S_1_ (tct) and S_2_ (tag).

The coding region of *F. diqingensis* mitogenome (GenBank accession number: PP083946) is 16,153 bp in size (the non-coding region is not complete, which will not be discussed in this article) (Figure 2). The nucleotide composition and skew are shown in Table 1. Among the 37 genes, 4 PCGs (*nad1*, *nad4*, *nad4L*, and *nad5*), 8 tRNA genes (*trnY*, *trnC*, *trnQ*, *trnV*, *trnL_1_* (*tag*), *trnP*, *trnH*, and *trnF*), and 2 rRNA genes (*rrnL* and *rrnS*) were encoded on the N-chain, while the remaining 23 genes (*nad2*, *cox1*, *cox2*, *atp8*, *atp6*, *cox3*, *nad3*, *nad6*, *cob*, *trnI*, *trnM*, *trnW*, *trnL_2_* (*taa*), *trnK*, *trnD*, *trnG*, *trnA*, *trnR*, *trnN*, *trnS_1_* (*tct*), *trnE*, *trnS_2_* (*tga*), and *trnT*) were encoded on the J-chain. The AT-skew of the *F. diqingensis* mitogenomes was −0.027, and the GC-skew was −0.228. There are a total of 13 intergenic spacer regions (106 bp), with the largest intergenic spacer region being 32 bp, followed by 21 bp. There are 11 overlap regions (28 bp), with the largest overlap region being 7 bp and the smallest overlap region being 1 bp.

**Table 1:** Organization of the *Frontopsylla diqingensis* mitogenome.

Name	Strand	Position	Length (bp)	Intergenic nucleotide	Nucleic acid		Codon		Anti-codon	AT-skew
					AT (%)	GC (%)	Start	Stop		
*Frontopsylla* sp.			16,153		75.5	24.6				−0.027
PCGs	J		6,846		73.9	26.1				−0.142
PCGs	N		4,275		77.3	22.7				−0.137
*trnI*	J	1,235 − 1,297	63						GAU	
*trnQ*	N	1,311-1,379	69	13					UUG	
*trnM*	J	1,379-1,446	68	-1					CAU	
*nad2*	J	1,447-2,455	1,009		79.8	20.2	ATT	T		−0.163
*trnW*	J	2,456-2,520	65						UCA	
*trnC*	N	2,520-2,581	62	−1					GCA	
*trnY*	N	2,582-2,644	63						GUA	
*cox1*	J	2,642-4,177	1,536	−3	67.7	32.3	ATC	TAA		−0.15
*trnL_2_(taa)*	J	4,182-4,245	64	4					UAA	
*cox2*	J	4,247-4,927	681	1	74	26.0	ATG	TAA		−0.048
*trnK*	J	4,930-4,999	70	2					CUU	
*trnD*	J	4,999-5,066	68	−1					GUC	
*atp8*	J	5,085–5,246	162	18	88.9	11.1	ATA	TAA		−0.014
*atp6*	J	5,240–5,914	675	−7	74.8	25.2	ATG	TAA		−0.141
*cox3*	J	5,914-6,696	783	−1	68.4	31.5	ATG	TAA		−0.119
*trnG*	J	6,697-6,758	62						UCC	
*nad3*	J	6,762-7,109	348	3	76.1	23.9	ATA	TAG		−0.253
*trnA*	J	7,108-7,172	65	−2					UGC	
*trnR*	J	7,171-7,234	64	−2					UCG	
*trnN*	J	7,256-7,320	65	21					GUU	
*trnS_1_(tct)*	J	7,321-7,389	69						UCU	
*trnE*	J	7,390-7,454	65						UUC	
*trnF*	N	7,453-7,521	69	−2					GAA	
*nad5*	N	7,522-9,237	1716		78.4	21.7	ATA	TAA		−0.105
*trnH*	N	9,242-9,303	62	4					GUG	
*nad4*	N	9,304-10,639	1,336		76.1	23.9	ATG	T		−0.150
*nad4L*	N	10,633-10,926	294	−7	81.7	18.4	ATG	TAA		−0.092
*trnT*	J	10,929-10,993	65	2					UGU	
*trnP*	N	10,994-11,057	64						UGG	
*nad6*	J	11,060-11,572	513	2	86.0	14.1	ATT	TAA		−0.188
*cob*	J	11,572 − 12,711	1,140	-1	72.2	27.8	ATG	TAA		−0.147
*trnS_2_(tga)*	J	12,715-12,780	66	3					UGA	
*nad1*	N	12,813-13,742	930	32	75.5	24.5	ATG	TAA		−0.194
*trnL_1_(tag)*	N	13,744-13,805	62	1					UAG	
*rrnL*	N	13,806-15,109	1,304		81.9	18.2				
*trnV*	N	15,110-15,176	67						UAC	
*rrnS*	N	15,177-15,965	789		80.1	19.9				

### 3.2 Mitogenome composition of *F. diqingensis*

The total length of the 13 protein-coding genes was 11,121 bp, the longest was *nad5* (1,716 bp), and the shortest was *atp8* (162 bp). All 13 protein-coding genes use the typical ATN as the start codon (ATG: 7, ATA: 3, ATT: 2, and ATC: 1), and the stop codons are TAA, TAG, and incomplete T (Table 1). The 13 protein-coding genes of *F. diqingensis* encode a total of 3,707 amino acids. The most frequently used code was UUA (RSCU = 4.46), and the lowest frequency used code was CCG (RSCU = 0.1) (Supplementary Table S4). The average length of 22 tRNAs was 65.3 ± 2.59 bp, and the size of a single gene was 62–70 bp. The secondary structure of tRNA is shown in Supplementary Figure S1. Among the 22 tRNA genes, except for *trnS_1_* (tct) lacking D-arm, all other tRNA genes formed typical cloverleaf structures. In addition to the typical Waston-Crick pairing (A-T, G-C), a total of 23 mismatches (G-T: 9, T-G: 7, T–T: 5, and A-A: 2) occurred in the mitogenome folding process of *F. diqingensis*, of which *trnH* mismatch was the most occurred (4 times) (Supplementary Figure S1). The total length of the two rRNAs was 2093 bp, both located on the N chain. The AT content of *rrnL* was 81.8%, and the AT content of *rrnS* was 80.1%.

### 3.3 The mitogenomes of different species in the subfamily Amphipsyllinae

*F. diqingensis*, *F. spadix*, and *P. custodis* showed high AT (75.5, 78.8, and 76.8%) contents. The AT-skew of *F. diqingensis* was −0.027, and the GC-skew was −0.228. The AT-skew of *F. spadix* was −0.036 and the GC-skew was −0.214. The AT-skew of *P. custodis* was −0.008, and the GC-skew was −0.259. The AT content of the first, second, and third codons of protein-coding genes of three species of flea was analyzed, and it was found that the AT content in the first and second codons was much smaller than that in the third codon. It shows that the third codon of the three species in Amphipsyllinae prefers to use A and T bases (Table 2). The protein-coding genes of three species use the typical ATN as the start codon, with ATG being used most frequently (Supplementary Table S5). Except for *nad2* and *nad4* with incomplete T or TA as the stop codon, the remaining 11 protein-coding use the typical TAN as the stop codon, with TAA used most frequently (Supplementary Table S5).

**Table 2:** Nucleotide composition of 3 species of the subfamily Amphipsyllinae.

Species	Gene	Size(bp)	T (%)	C (%)	A (%)	G (%)	AT (%)	AT-skew	GC-skew
*Frontopsylla diqingensis*	All genes	16,153	38.8	15.1	36.7	9.5	75.5	−0.027	−0.228
	First codon position	3,707	36	11.8	34.7	17.5	70.7	−0.019	0.196
	Second codon position	3,707	47.9	16.9	21.1	14.1	69	−0.389	−0.091
	Third codon position	3,707	44.6	8.1	41.3	6	85.9	−0.039	−0.148
	PCGs	11,121	42.9	12.2	32.4	12.5	75.3	−0.14	0.012
	tRNA	1,437	40.1	9	39.4	11.5	79.5	−0.009	0.119
	rRNA	2,093	39.4	5.9	41.8	12.9	81.2	0.029	0.376
*F. spadix*	All genes	15,085	40.8	12.8	38	8.3	78.8	−0.036	−0.214
	First codon position	3,714	36.8	11.1	35.1	16.9	71.9	−0.024	0.207
	Second codon position	3,714	47.9	16.9	21.1	14.1	69	−0.39	−0.09
	Third codon position	3,714	47	5.3	44.5	3.2	91.5	50.2	−0.027
	PCGs	11,142	43.9	11.1	33.6	11.4	77.5	−0.134	0.013
	tRNA	1,439	39.6	8.8	40.6	11	80.2	0.012	0.109
	rRNA	2,067	40.1	5.8	42.3	11.8	82.4	0.026	0.341
*Paradoxopsyllus custodis*	All genes	15,375	38.7	14.6	38.1	8.6	76.8	−0.008	−0.259
	First codon position	3,703	36.8	11.7	34.1	17.4	70.9	−0.038	0.193
	Second codon position	3,703	47.6	17.6	20.7	14.2	68.3	−0.395	−0.107
	Third codon position	3,703	43.8	8.3	41.5	6.4	85.3	−0.028	−0.128
	PCGs	11,109	42.7	12.5	32.1	12.6	74.8	−0.143	0.004
	tRNA	1,432	39.5	8.4	40.8	11.3	80.3	0.016	0.149
	rRNA	2,070	40.2	6.3	40.1	13.4	80.3	−0.001	0.361

The tRNA secondary structures of *F. diqingensis*, *F. spadix*, and *P. custodis* in Amphipsyllinae have little difference among different species, and all of them form typical cloverleaf structure (except *trnS_1_* (tct)). The 22 tRNAs had 1,458 sites, of which 1,312 were conserved, accounting for 90.0% of the total sites. There were 146 variable sites, accounting for 10.0% of the total sites. There were 14 mutation sites of *trnC* and 1 mutation site of *trnQ* (Supplementary Table S6). The majority of these mutation sites were at the junction of arms and rings (Supplementary Figure S2).

The nucleotide diversity index (*π*) of 13 protein-coding genes in Amphipsyllinae was 0.3842. There were 3,991 variable sites (V), 7,147 conserved sites (C), and 3,890 singleton sites (S).

### 3.4 The relative synonymous codon usage (RSCU) of the subfamily Amphipsyllinae

**Figure 3:**
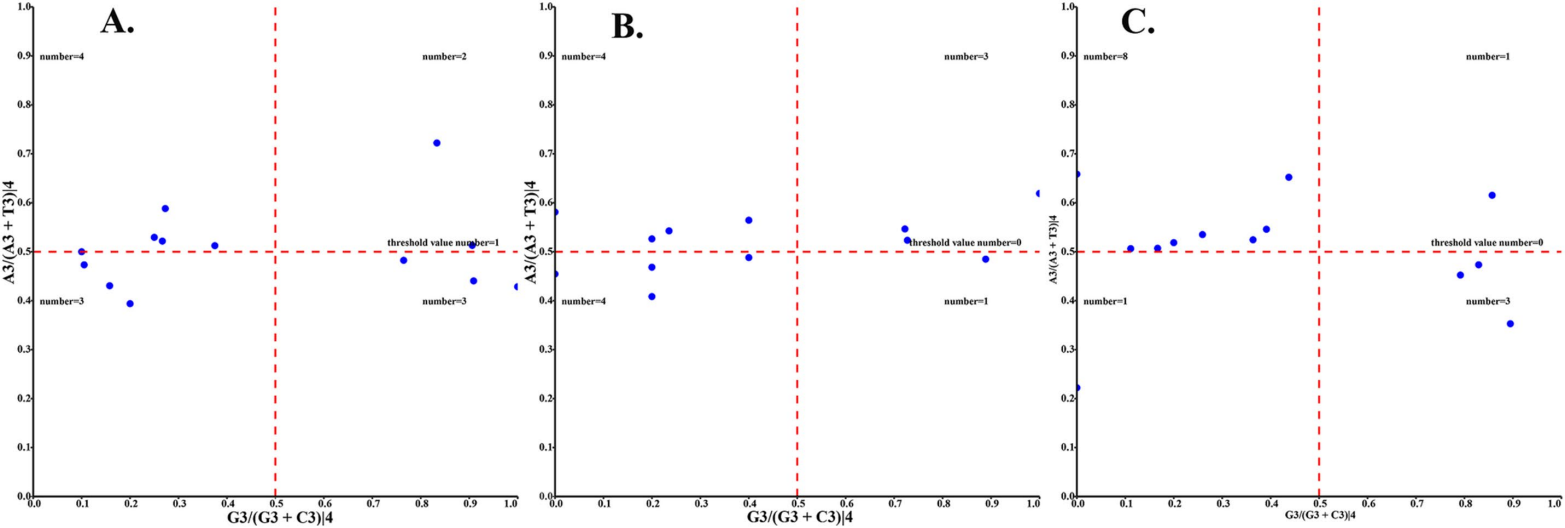
Codon usage preference analysis of PCGs in the subfamily Amphipsyllinae by PR2, the *y*-axis represents the content of A/(A + T) at the third position of the codon, and the *x*-axis represents the content of G/(G + C) at the third position of the codon. **(A)** *Frontopsylla diqingensis*; **(B)** *Frontopsylla spadix*; **(C)** *Paradoxopsyllus custodis* (A blue dot indicates a protein-coding gene).

**Figure 4:**
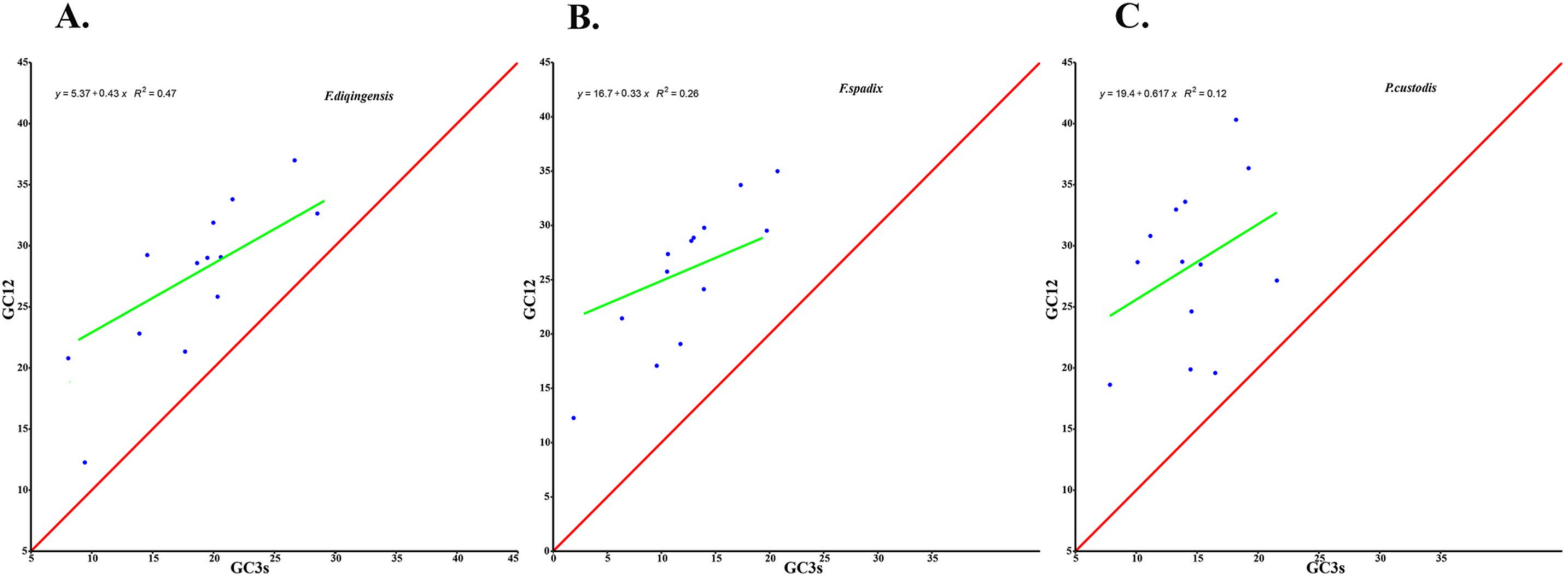
Codon usage preference analysis of PCGs in the subfamily Amphipsyllinae by neutral curve. The *y*-axis represents the average GC content of the first base and the second base (GC12) of the codon, and the *x*-axis represents the content of G and C at the third position of codon. (**A)**. *Frontopsylla diqingensis*; (**B)***. Frontopsylla spadix*; (**C)***. Paradoxopsyllus custodis.* (A blue dot indicates a protein-coding gene).

**Figure 5:**
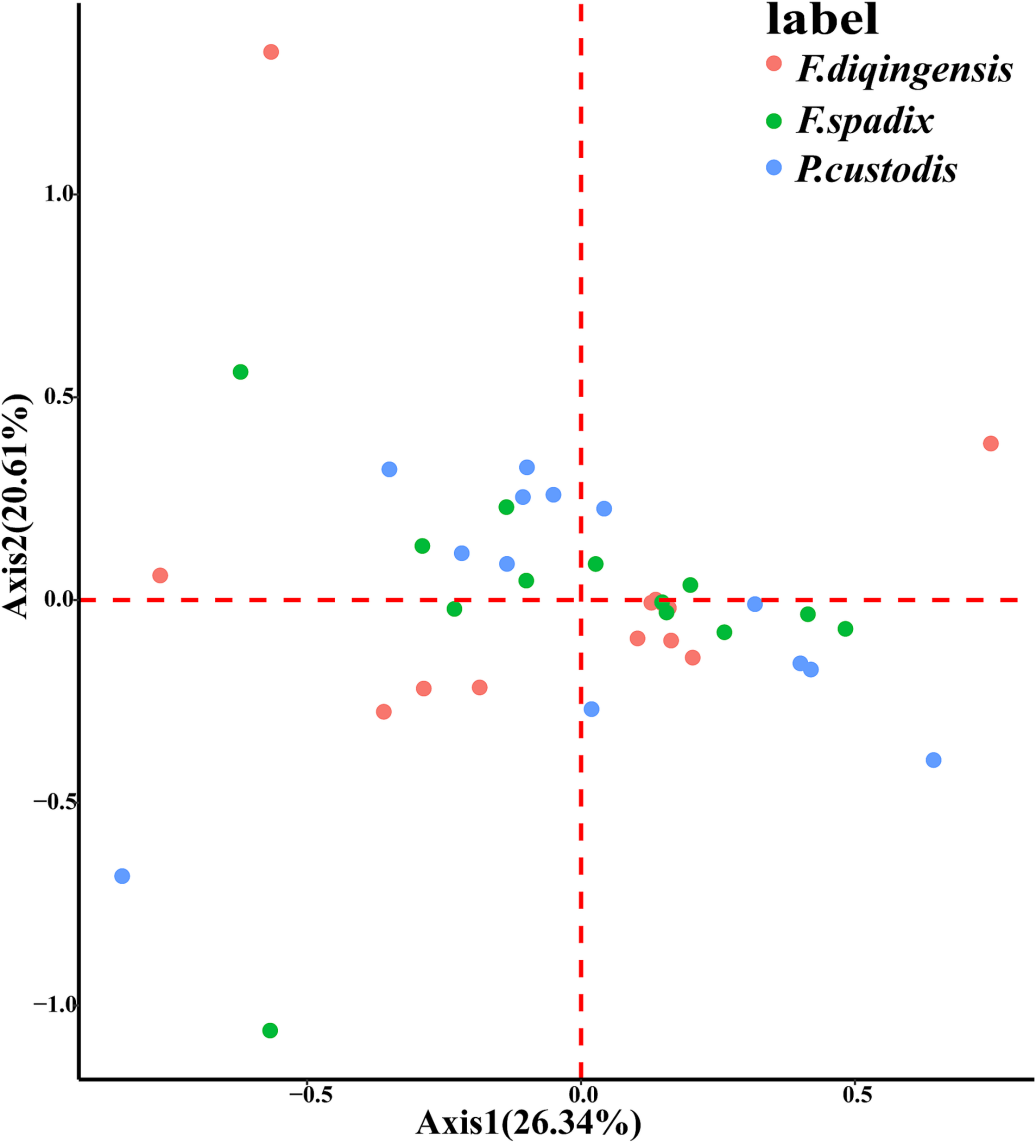
Correspondence analysis (COA) of PCGs in the subfamily Amphipsyllinae.

The relative synonymous codon usage of three species of Amphipsyllinae was analyzed and found that the codon usage preference patterns of *F. diqingensis*, *F. spadix* and *P. custodis* were very similar (Supplementary Table S4, Supplementary Figure S3). *F. diqingensis*, *F. spadix*, and *P. custodis* had 26, 27, and 27 high-frequency codons (RSCU >1), respectively. It can be seen from RSCU that there is a specific preference for codon usage, but the reason for its preference is not apparent. We use parity rule 2 (PR2), effective number of codon (ENC), neutrality curve, and corresponding analysis (COA) to analyze the reasons that affect codon usage preference. PR2 showed that mutation, selection, and other factors affected codon usage through qualitative analysis and found that protein-coding genes of three species of fleas were not located at the central site (0.5, 0.5). Among them, *F. diqingensis* and *F. spadix* have a T/C preference, and *P. custodis* has an A/C preference (Figure 3). The ENC values of protein-coding genes of 3 species in Amphipsyllinae ranged from 30.11 to 46.54. The majority of them were below the standard curve (the *atp8* gene is not included because CodonW calculates the NC value based on the principle of ENC (50) calculation, and when the gene does not contain amino acids with eight synonymous genes, the ENC value of this sequence is not calculated). Among them, 13 protein-coding genes Nc ≤ 35 have a total of 15 genes, *F. diqingensis* has 4 genes, *F. spadix* has 9 genes, and *P. custodis* has 2 genes (Supplementary Figure S4). In the neutrality curve, GC12 of three species has no significant linear relationship with GC3s (the coefficient of determination R^2^ is used mainly to quantify the strength of the linear relationship between two variables. The closer the R^2^ value is to 1, the stronger the linear relationship between GC12 and GC3s; the closer the R^2^ value is to 0, the weaker the linear relationship between GC12 and GC3s). Therefore, the absolute value of the regression coefficient cannot represent the degree of mutation pressure on protein-coding genes in the species (Figure 4). In the COA analysis, three species of fleas are scattered together. There is no situation in which one species is clustered alone, indicating that *F. diqingensis*, *F. spadix*, and *P. custodis* have similar codon usage preferences (Figure 5).

### 3.5 The evolutionary relationships of the subfamily Amphipsyllinae

**Figure 6:**
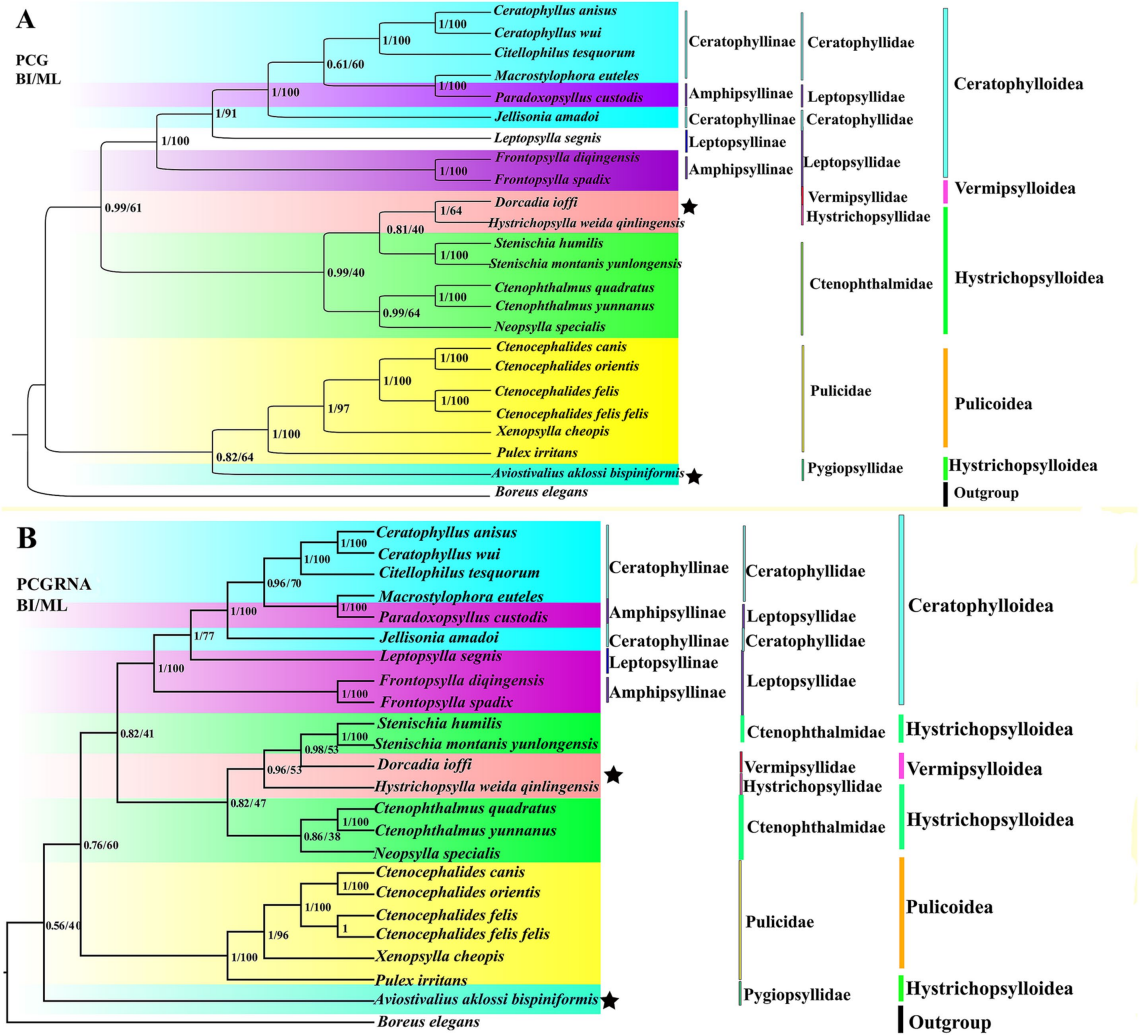
Phylogenetic relationship of the order Siphonaptera was reconstructed by Bayesian inference (BI) and maximum likelihood (ML) method. (A): PCG; (B): PCGRNA). (“★” indicates topological inconsistency).

We obtained four trees from two concatenated data matrices (PCG and PCGRNA) and two tree-construction methods (ML and BI) (Figure 6). Four trees are highly similar; the difference is that the taxonomic status of *Hystrichopsylla weida qinlingensis* Zhang et al. (1984), *Dorcadia ioffi* Smit (1953), and *Aviostivalius aklossi bispiniformis* Li and Wang (1958), is ambiguous. In Figure 6A, PCG: The 23 flea species are divided into two major lineages, forming three clades: (Pulicidae + Pygiopsyllidae) forms one clade, while ((Ceratophyllidae + Leptopsyllidae) + ((Vermipsyllidae + Hystrichopsyllidae) + Ctenophthalmidae)) forms two clades that are more closely related. In Figure 6B, PCGRNA: The 23 flea species are divided into two major lineages, forming three clades: Pygiopsyllidae form one clade, while ((Ceratophyllidae + Leptopsyllidae) + ((Vermipsyllidae + Hystrichopsyllidae) + Ctenophthalmidae)), and Pulicidae forms two clades that are more closely related. The families Leptopsyllidae, Ctenophthalmidae, and the subfamily Ceratophyllinae are paraphyletic groups. The families Pulicidae and Ceratophyllidae form a monophyletic group. The families Pulicidae and Pygiopsyllidae are closely related to the base of the phylogenetic tree. In Amphipsyllinae, *F. diqingensis* and *F. spadix* were clustered into one clade (BPP = 1, BSP = 100). *P. custodis* and *Macrostylophora euteles* Jordan *et* Rothschild, 1,911 were clustered into one clade (BPP = 1, BSP = 100). The families Ctenophthalmidae, Vermipsyllidae (one species), and Hystrichopsyllidae (one species) had low node support.

### 3.6 Divergence time estimation of the subfamily Amphipsyllinae

**Figure 7:**
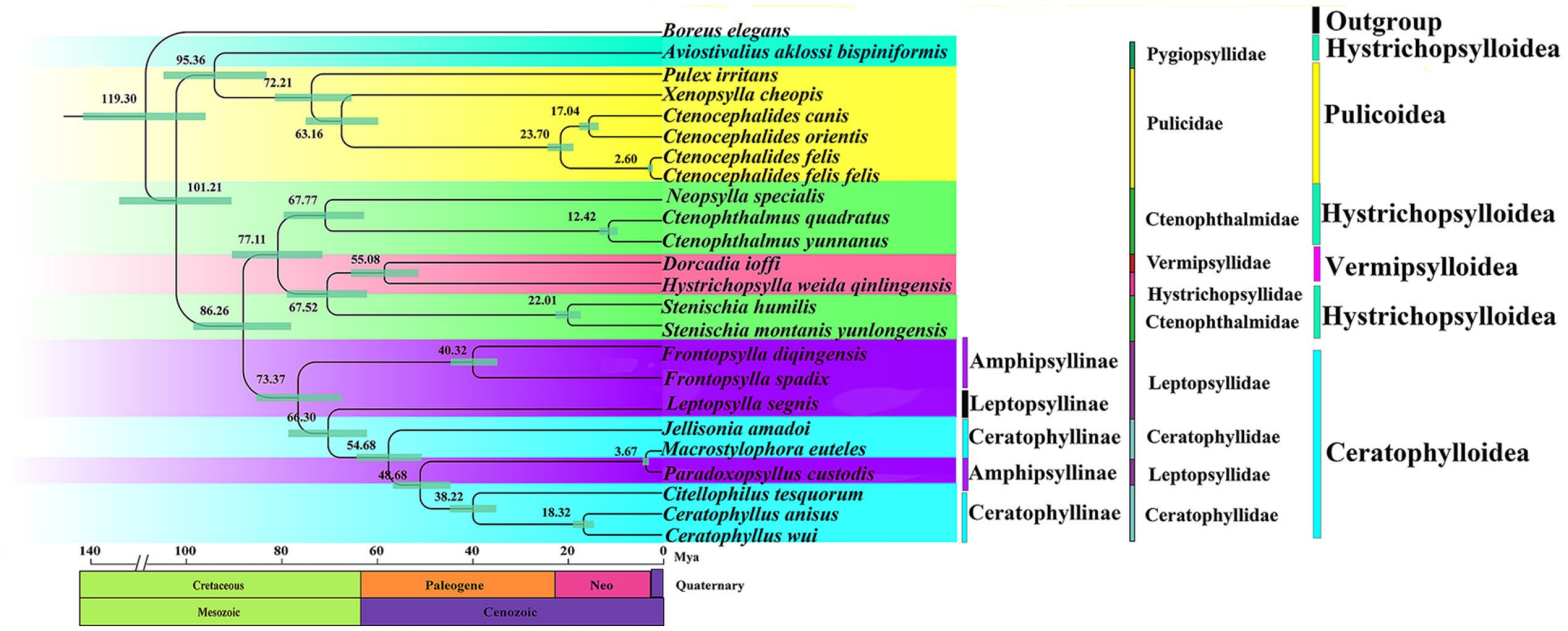
Divergence time estimation of various lineage in the order Siphonaptera (Node labels are the mean values of the estimated ages. The node bars indicate 95% confidence intervals).

According to the estimated divergence time, Siphonaptera originated in the Early Cretaceous around 119.3 Mya (the divergence times and confidence intervals for nodes/clades are shown in Supplementary Table S7). Clade 1 (Pygiopsyllidae and Pulicidae) begins to diverge from clades 2 (Ctenophthalmidae; Vermipsyllidae; and Hystrichopsyllidae) and 3 (Leptopsyllidae and Ceratophyllidae) at 101.21 Mya. Clade 2 (Ctenophthalmidae; Vermipsyllidae; and Hystrichopsyllidae) and Clade 3 (Leptopsyllidae and Ceratophyllidae) began to diverge at 86.26 Mya. The divergence time between Pygiopsyllidae and Pulicidae is approximately 95.36 Mya, and the relationship between Pygiopsyllidae and Pulicidae is closer. Amphipsyllinae diverged approximately between 73.37 and 40.32 Mya. The Ceratophyllidae family diverged most recently at 73.37 Mya, while *F. spadix* and *F. diqingensis* diverged at approximately 40.32 Mya. The majority of the intraordinal divergence into Siphonaptera’s extant lineages occurred after the K-Pg boundary (Figure 7).

## 4 Discussion

Morphological characteristics are the basis for systematic taxonomy. Accurate identification is essential for reconstructing phylogeny. The majority of the identification maps of flea species in the available data are hand-drawn and lack clear color maps. Therefore, this study presents a clear color morphology map to supplement the hand-drawn maps. Three species of Amphipsyllinae retained the ancestral pattern of mitochondrial gene arrangement of arthropods. AT-skew is negative and is mainly influenced by directional evolution pressure and asymmetric replication. The nucleotide diversity of 13 protein-coding genes in Amphipsyllinae was 0.3842, indicating that nucleotide diversity differences among Amphipsyllinae were significant, and showed high genetic diversity. Gene exchange between species was more frequent. Among them, the *nad4* and *nad6* genes were the two genes with the highest nucleotide diversity among the 13 protein-coding genes, indicating that these two genes have high nucleotide variability within Amphipsyllinae, and can be used as potential molecular markers for future exploration of population differentiation in Amphipsyllinae (Supplementary Figure S4).

**Figure 8:**
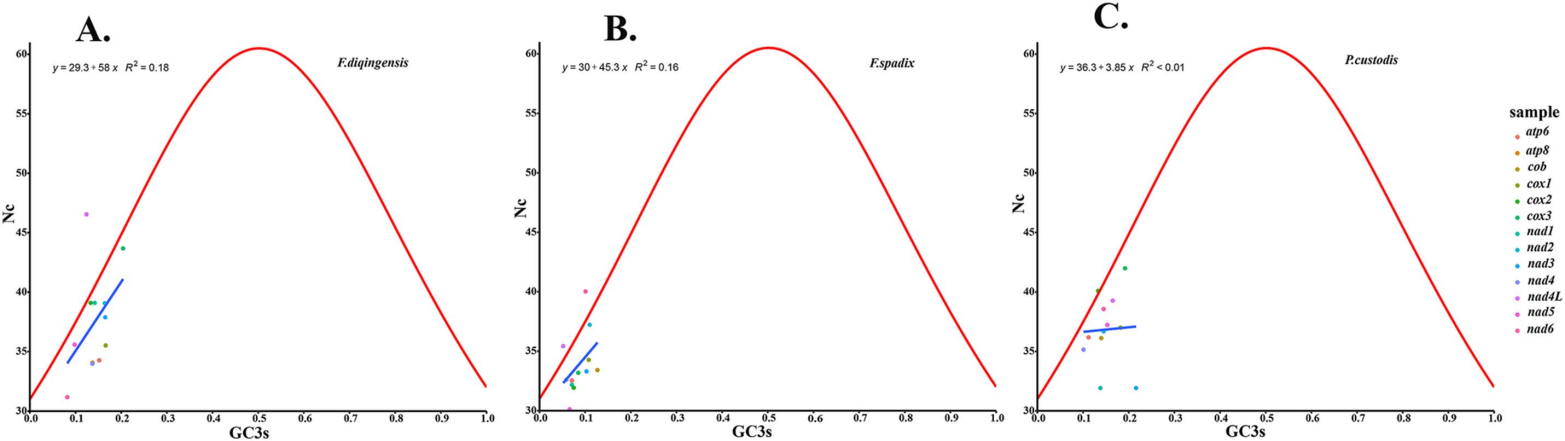
Codon usage preference analysis of PCGs in the subfamily Amphipsyllinae by ENC-plot, the *y*-axis represents the Nc value, and the *x*-axis represents the content of G and C at the third position of the codon. **(A)** *Frontopsylla diqingensis*; **(B)** *Frontopsylla spadix*; **(C)** *Paradoxopsyllus custodis.*

We analyzed the relative synonymous codon usage (RSCU) of 13 protein-coding genes in three species of Amphipsyllinae and evaluated the synonymous codon usage bias (Supplementary Table S4, Supplementary Figure S3). An RSCU >1.6 indicates an overrepresented codon, while an RSCU >1 indicates a strong preference for that codon. An RSCU of 1 indicates no preference, meaning all codons are used equally, and an RSCU <1 indicates a weak preference (51, 52). An RSCU of 0 indicates that the codon has not been used. It was noted that the high-frequency codons predominantly end in A or U, while the codons ending with G or C were either rarely used or not used at all. Specific codons always tend to form codon clusters or repeats (53). There are also unique species preferences for G/C-ending codons (54, 55). We used COA analysis (Figure 5), PR2 (Figure 3), ENC-plot (Figure 8), and neutral curve analyses (Figure 4) to further explore the causes of codon usage bias. In the ENC-plot analysis, there were 15 genes with Nc < 35 in three species of fleas, with Nc < 35 indicating a significant preference for codon usage (56). At the same time, the Nc value is also an essential index for evaluating the expression of endogenous genes. The codon preference of highly expressed genes is greater, and their Nc values are smaller. On the other hand, lowly expressed genes contain a greater variety of rare codons and have larger Nc values (50). Only a few genes in three species of fleas fall above the standard curve, and the majority of the genes fall below it, indicating that the majority of the genes have been influenced by natural selection during evolution. No genes were located at the coordinates (0.5, 0.5) for three species of fleas in the PR2 analyses, suggesting that these genes have been influenced mainly by selection during evolution. Because GC content is usually determined by the mutation process of the whole genome (57), it often reflects the strength of directional mutation pressure (58). In the case of mutation pressure, the mutation at the third position of the codon usually does not alter the type of the amino acid, which is a synonymous mutation. Mutations occurring at the first and second codons are non-synonymous mutations, which affect the function and activity of related genes, but these mutations do not happen frequently. Therefore, the probability of mutation at the third codon position is much greater than that at the first and second codon positions. In the neutral curve analysis, the GC12 and GC3s of protein-coding genes of three species of fleas have no significant linear relationship, and the genes were scattered, indicating that these genes were mainly affected by selection during evolution. In summary, it can be deduced that the main reason for codon bias of 13 protein-coding genes in Amphipsyllinae was selection pressure. Additionally, base composition (59), nature of amino acids (aromatic and hydrophobic) (60), and codon context (61) might also be influenced by codon usage preference. There are more generally accepted theories, such as the “energy compensation” theory, which thought that the synthesis of the G + C base pair required more energy and nitrogen than the A + T base pair in nucleotide synthesis (62). The “selection-mutation-drift” theory suggested that mutations were predisposed, and natural selection reflected codon usage preference in the evolutionary process (63). These theories need to be studied further.

Phylogenetic trees were reconstructed using both BI and ML methods, based on two concatenated data matrices from 23 species of Siphonaptera. The majority of the species in the ingroup are clustered together in the same family or genus, and the taxonomic status is clear. Ceratophyllinae, Leptopsyllinae, and Amphipsyllinae are clustered into one clade. *F. spadix* and *F. diqingensis* clustered into one clade (BPP = 1, BSP = 100). Consistent with traditional taxonomy. To one’s surprise, *P. custodis* and *M. euteles* of different subfamilies were clustered together (BPP = 1, BSP = 100). We further alignment and found the mtDNA sequence of *P. custodis* and *M. euteles* are highly similar (98.8%), *cox1* was 98.6%, *cob* was 98.8%, *rrnL* was 99.5%, and *rrnS* was 99.8%. This suggests a possible error in the author’s uploaded sequences, as *P. custodis* and *M. euteles* sequences from GenBank may actually represent the same species. As shown in Figure 6, Amphipsyllinae was monophyletic (currently, with relatively poor species, it could affect the accuracy of phylogenetic estimation). Ceratophyllinae and Ctenophthalmidae are paraphyletic. Pulicidae and Pygiopsyllidae are located at the base of the phylogenetic tree, indicating that Pulicidae and Pygiopsyllidae are the earliest divergence groups among the selected 23 species. The divergence time tree (Figure 7) showed that Pygiopsyllidae and Pulicidae were the first groups to diverge (95.36 Mya). However, Wu (14) suggested that the family Ceratophyllidae should be placed above the family Pulicidae (which includes the previous family Tungidae), and additional data are needed to confirm this. Vermipsyllidae, Hystrichopsyllidae, and Ctenophthalmidae were clustered together (BPP = 0.81/96, BSP = 0.40/53), although the node had weak support, except for PCGRNA (BI), which showed strong node support, in line with previous findings (64–66). This showed the taxonomic status of the superfamily Hystrichopsylloidea is in a state of confusion. Morphologically, the three families are quite different, but at the molecular level, they belong to the same evolutionary clade, which may be the reason why there are too few species, and it is hoped that more species will be added to resolve their taxonomic status in the future. Whether the superfamilies Vermipsylloidea and Hystrichopsylloidea are separated from the superfamily Ceratophylloidea to become two separate families remains to be investigated (14).

The divergence time of various lineages in Siphonaptera was estimated and showed that it comprises two sequentially derived lineages, of which Pulicidae and Pygiopsyllidae diverged earlier. The subfamily Amphipsyllinae is the earliest diverging of the 3 subfamilies (Ceratophyllinae, Leptopsyllinae, and Amphipsyllinae), which is also reflected in its higher nucleotide diversity index (Figure 7). Figure 7 shows that the common ancestor of the extant fleas diverged during the Cretaceous. The majority of intrordinal divergence into extant flea lineages occurred after the K-Pg boundary, consistent with Zhu et al. (1). However, this timeline is far from the Mesozoic fossil “dinosaur fleas” identified by Huang et al. in Northeast China, as well as from the pterosaur to mammal hosts. Two fleas (100-125Mya) from the Cretaceous period were reported by some scholars, but the specimens were lost, and these records could not be confirmed (67). Even the attribution of certain Mesozoic fossils to Siphonaptera provides some interesting scenarios of flea evolutionary history. For example, Rasnitsyn (2002) considers these three fossils (*Strashila incredibilis*, *Saurophthirus longipes*, and *Tawinia australis*) together as “pre-fleas” because of the prominent lower forehead and relatively short antennae (68). We did not support the aforementioned opinions. Our findings supported the study by Zhu et al. (1). First, dinosaur fleas are morphologically quite different from extant fleas, and the earliest definitive fossil specimen of a flea has so far been found in the Eocene (*Palaeopsylla* spp. in Baltic Amber) (4). Second, there were conflicts in geographical location (Northeast China belongs to Laurasia, and fleas originated in Gondwana). Finally, Zhu et al. (1) used 205 species of fleas from 16 families to prepare DNA sequence datasets of mitogenomes, nuclear protein coding, and ribosomal genes for constructing phylogenetic trees and divergence time trees. Combined with the ancestral host associations and geographical distribution, it is concluded that the earliest differentiation is Macropsyllidae (Late Cretaceous). In summary, we supported that the common ancestors of fleas diverged in the Cretaceous. We cannot rule out errors in divergence time estimation, because of the initial use of mitogenomes and the scarcity of exact fossils of fleas. Mitochondrial genomes evolve at a faster rate than nuclear genomes, making them more effective for studies at lower taxonomic ranks. However, they have limitations when applied to studies at higher taxonomic ranks. The rapid evolution rate may lead to long-branch attraction (LBA) artifacts, which would affect the accuracy of the phylogeny. The evolutionary history and diversification of the tribe can be better resolved by increasing species representation and geographic coverage.

## 5 Conclusion

In this study, we sequenced the mitogenome of *F. diqingensis* for the first time. The composition of mitogenomes in three species from Amphipsyllinae differed among species. The phylogenetic tree supported that the majority of the species in the ingroup are clustered together in the same family or genus. Ceratophyllinae and Ctenophthalmidea are paraphyletic groups, while Pulicidae and Ceratophyllidae are monophyletic. We concluded that *P. custodis* and *M. euteles* from GenBank are the same species by phylogenetic trees and sequence alignment, and supported the monophyly of Amphipsyllinae. Divergence time estimation showed that the common ancestor of fleas diverged in the Cretaceous. Amphipsyllinae begins to diverge at approximately 73.37–40.32 Mya. The majority of the species of the intraordinal divergence into extant lineages occurred after the K-Pg boundary. This study provides new insights into the evolutionary history and taxonomy of Siphonaptera.

## Data Availability

The datasets presented in this study can be found in online repositories. The names of the repository/repositories and accession number(s) can be found below: https://www.ncbi.nlm.nih.gov/nuccore/PP083946.1/.
