# Supplementary material for: Phylogeny and divergence time estimation of the subfamily Amphipsyllinae based on the Frontopsylla diqingensis mitogenome
Source: Front Vet Sci. 2024 Dec 11;11:1494204. doi: 10.3389/fvets.2024.1494204 (PMC11668791; doi:10.3389/fvets.2024.1494204)
Supplement: Supplementary file 1 [file Table_1.DOCX]

Supplementary Material

**Table S1.** Species information used for phylogenetic and divergence time analyses in this study

| Family | Species | GenBank number |
| --- | --- | --- |
| Pulicidae | *Ctenocephalides felis* | MT594468 |
|  | *Ctenocephalides felis felis* | MW420044 |
|  | *Ctenocephalides orientis* | NC 073009 |
|  | *Ctenocephalides canis* | ON 109770 |
|  | *Pulex irritans* | ON 100828 |
|  | *Xenopsylla cheopis* | MW310242 |
| Hystrichopsyllidae | *Hystrichopsylla weida qinlingensis* | NC 042380 |
| Pygiopsyllidae | *Aviostivalius aklossi bispiniformis* | OR 774970 |
| Ctenophthalmidae | *Neopsylla specialis* | NC 073019 |
|  | *Stenischia humilis* | NC 073020 |
|  | *Stenischia montanis yunlongensis* | OR780663 |
|  | *Ctenophthalmus yunnanus* | OR780664 |
|  | *Ctenophthalmus quadratus* | NC 072692 |
| Leptopsyllidae | *Paradoxopsyllus custodis* | OQ627398 |
|  | *Frontopsylla spadix* | NC073018 |
|  | *Frontopsylla diqingensis* | PP083946 |
|  | *Leptopsylla segnis* | NC 072691 |
| Ceratophyllidae | *Citellophilus tesquorum* | PP418872 |
|  | *Ceratophyllus wui* | NC040301 |
|  | *Jellisonia amadoi* | NC022710 |
|  | *Ceratophyllus anisus* | NC073017 |
|  | *Macrostylophora euteles* | OR774969 |
| Vermipsyllidae | *Dorcadia ioffi* | NC 036066 |
| Boreidae | *Boreus elegans* | HQ696579 |

**Table S2：**The partition strategies and models inferred by ModelFinder and used in this study (PCG)

| Datasets | Subset partitions | Best model |
| --- | --- | --- |
| PCG (BI) | P1: (atp6_codon1, cox1_codon1, cox2_codon1, cox3_codon1, cytb_codon1) | GTR+F+I+G4 |
|  | P2: (atp6_codon2, cox1_codon2, cox2_codon2, cox3_codon2, cytb_codon2, nad1_codon2, nad2_codon2, nad3_codon2, nad4L_codon2, nad4_codon2, nad5_codon2, nad6_codon2) | GTR+F+I+G4 |
|  | P3: (atp6_codon3, cox1_codon3, cox2_codon3) | HKY+F+I+G4 |
|  | P4: (atp8_codon1, atp8_codon2, nad2_codon1, nad3_codon1, nad6_codon1) | GTR+F+I+G4 |
|  | P5: (atp8_codon3, cox3_codon3, cytb_codon3, nad3_codon3, nad6_codon3) | GTR+F+G4 |
|  | P6: (nad1_codon1, nad4L_codon1, nad4_codon1, nad5_codon1) | GTR+F+I+G4 |
|  | P7: (nad1_codon3, nad2_codon3, nad4L_codon3, nad4_codon3) | HKY+F+I+G4 |
|  | P8: (nad5_codon3) | GTR+F+G4 |
|  |  |  |
| PCG (ML) | P1: (atp6_codon1, cox1_codon1, cox2_codon1, cox3_codon1, cytb_codon1) | GTR+F+I+G4 |
|  | P2: (atp6_codon2, cox1_codon2, cox2_codon2, cox3_codon2, cytb_codon2, nad1_codon2, nad2_codon2, nad3_codon2, nad4L_codon2, nad4_codon2, nad5_codon2, nad6_codon2) | GTR+F+R3 |
|  | P3: (atp6_codon3, cox1_codon3, cox2_codon3) | TPM2u+F+I+G4 |
|  | P4: (atp8_codon1, atp8_codon2, nad2_codon1, nad3_codon1, nad6_codon1) | TIM2+F+I+G4 |
|  | P5: (atp8_codon3, cox3_codon3, cytb_codon3, nad3_codon3, nad6_codon3) | TIM2+F+G4 |
|  | P6: (nad1_codon1, nad4L_codon1, nad4_codon1, nad5_codon1) | TVM+F+I+G4 |
|  | P7: (nad1_codon3, nad2_codon3, nad4L_codon3, nad4_codon3) | TN+F+I+I+R3 |
|  | P8: P8: (nad5_codon3) | TIM+F+R2 |

**Table S3：**The partition strategies and models inferred by ModelFinder and used in this study (PCGRNA)

| Datasets | Subset partitions | Best model |
| --- | --- | --- |
| PCGRNA(BI) | P1: (atp6_codon1, cox1_codon1, cox2_codon1, cox3_codon1, cytb_codon1) | GTR+F+I+G4 |
|  | P2: (atp6_codon2, cox1_codon2, cox2_codon2, cox3_codon2, cytb_codon2, nad1_codon2, nad2_codon2, nad3_codon2, nad4L_codon2, nad4_codon2, nad5_codon2, nad6_codon2) | GTR+F+I+G4 |
|  | P3: (atp6_codon3, cox1_codon3, cox2_codon3) | HKY+F+I+G4 |
|  | P4: (atp8_codon1, atp8_codon2, nad2_codon1, nad3_codon1, nad6_codon1) | GTR+F+I+G4 |
|  | P5: (atp8_codon3, cox3_codon3, cytb_codon3, nad3_codon3, nad6_codon3) | GTR+F+G4 |
|  | P6: (nad1_codon1, nad4L_codon1, nad4_codon1, nad5_codon1) | GTR+F+I+G4 |
|  | P7: (nad1_codon3, nad2_codon3, nad4L_codon3, nad4_codon3) | HKY+F+I+G4 |
|  | P8: (nad5_codon3) | GTR+F+G4 |
|  | P9: (rrnL, rrnS) | GTR+F+I+G4 |
|  |  |  |
| PCGRNA(ML) | P1: (atp6_codon1, cox1_codon1, cox2_codon1, cox3_codon1, cytb_codon1) | GTR+F+I+G4 |
|  | P2: (atp6_codon2, cox1_codon2, cox2_codon2, cox3_codon2, cytb_codon2, nad1_codon2, nad2_codon2, nad3_codon2, nad4L_codon2, nad4_codon2, nad5_codon2, nad6_codon2) | GTR+F+R3 |
|  | P3: (atp6_codon3, atp8_codon3, cox1_codon3, cox2_codon3, cox3_codon3, cytb_codon3, nad3_codon3, nad6_codon3) | TIM2+F+I+G4 |
|  | P4: (atp8_codon1, atp8_codon2, nad2_codon1, nad3_codon1, nad6_codon1) | TIM2+F+I+G4 |
|  | P5: (nad1_codon1, nad4L_codon1, nad4_codon1, nad5_codon1) | GTR+F+I+G4 |
|  | P6: (nad1_codon3, nad2_codon3, nad4L_codon3, nad4_codon3) | TN+F+I+I+R3 |
|  | P7: (nad5_codon3) | TIM+F+G4 |
|  | P8:(rrnL, rrnS) | GTR+F+I+G4 |

**TABLE S4.** Codon usage of 3 species of Amphipsyllinae

| Amino acid | Codon | RSCU |  |  | Amino acid | Codon | RSCU |  |  |
| --- | --- | --- | --- | --- | --- | --- | --- | --- | --- |
|  |  | *F. d* | *F. s* | *P. c* |  |  | *F. d* | *F. s* | *P. c* |
| Phe | UUU | 1.73 | 1.81 | 1.74 | Thr | ACG | 0.11 | 0.06 | 0.08 |
|  | UUC | 0.27 | 0.19 | 0.26 | Ala | GCU | 2.14 | 2.21 | 2.07 |
| Leu | UUA | 4.46 | 4.75 | 4.4 |  | GCC | 0.58 | 0.35 | 0.55 |
|  | UUG | 0.16 | 0.14 | 0.29 |  | GCA | 0.96 | 1.38 | 1.14 |
|  | CUU | 0.66 | 0.7 | 0.55 |  | GCG | 0.32 | 0.06 | 0.23 |
|  | CUC | 0.17 | 0.07 | 0.13 | Tyr | UAU | 1.75 | 1.78 | 1.73 |
|  | CUA | 0.51 | 0.32 | 0.55 |  | UAC | 0.25 | 0.22 | 0.27 |
|  | CUG | 0.04 | 0.01 | 0.07 | His | CAU | 1.69 | 1.64 | 1.36 |
| Ile | AUU | 1.72 | 1.91 | 1.79 |  | CAC | 0.31 | 0.36 | 0.64 |
|  | AUC | 0.28 | 0.09 | 0.21 | Gln | CAA | 1.77 | 1.97 | 1.8 |
| Met | AUA | 1.74 | 1.86 | 1.73 |  | CAG | 0.23 | 0.03 | 0.2 |
|  | AUG | 0.26 | 0.14 | 0.27 | Asn | AAU | 1.74 | 1.7 | 1.66 |
| Val | GUU | 1.97 | 1.93 | 1.81 |  | AAC | 0.26 | 0.3 | 0.34 |
|  | GUC | 0.23 | 0.14 | 0.31 | Lys | AAA | 1.77 | 1.68 | 1.8 |
|  | GUA | 1.54 | 1.82 | 1.57 |  | AAG | 0.23 | 0.32 | 0.2 |
|  | GUG | 0.26 | 0.11 | 0.31 | Asp | GAU | 1.59 | 1.66 | 1.52 |
| Ser | UCU | 2.73 | 2.64 | 2.5 |  | GAC | 0.41 | 0.34 | 0.48 |
|  | UCC | 0.43 | 0.31 | 0.39 | Glu | GAA | 1.48 | 1.77 | 1.55 |
|  | UCA | 1.85 | 2.04 | 2.18 |  | GAG | 0.52 | 0.23 | 0.45 |
|  | UCG | 0.07 | 0.02 | 0.11 | Cys | UGU | 1.64 | 1.83 | 1.71 |
|  | AGU | 0.5 | 0.56 | 0.51 |  | UGC | 0.36 | 0.17 | 0.29 |
|  | AGC | 0.14 | 0.09 | 0.21 | Trp | UGA | 1.77 | 1.9 | 1.73 |
|  | AGA | 1.88 | 2.22 | 1.93 |  | UGG | 0.23 | 0.1 | 0.27 |
|  | AGG | 0.41 | 0.11 | 0.17 | Arg | CGU | 0.62 | 1.08 | 0.83 |
| Pro | CCU | 2.23 | 2.44 | 2.27 |  | CGC | 0.31 | 0.08 | 0.23 |
|  | CCC | 0.52 | 0.33 | 0.6 |  | CGA | 2.77 | 2.85 | 2.19 |
|  | CCA | 1.15 | 1.17 | 1.01 |  | CGG | 0.31 | 0 | 0.75 |
|  | CCG | 0.1 | 0.07 | 0.13 | Gly | GGU | 0.81 | 0.85 | 0.71 |
| Thr | ACU | 1.79 | 1.96 | 2.06 |  | GGC | 0.17 | 0.19 | 0.17 |
|  | ACC | 0.45 | 0.11 | 0.44 |  | GGA | 1.92 | 2.31 | 1.89 |
|  | ACA | 1.65 | 1.88 | 1.42 |  | GGG | 1.1 | 0.64 | 1.22 |

Note: *F.d* represents *Frontopsylla diqingensis*, *F.s* represents *Frontopsylla spadix*, *P.c* represents *Paradoxopsyllus custodis*

**TABLE S5.** Comparison of start and stop codons in 3 species of the subfamily Amphipsyllinae.

|  | Start | | | Stop | | |
| --- | --- | --- | --- | --- | --- | --- |
| Gene | *F. diqingensis* | *F. spadix* | *P. custodis* | *F. diqingensis* | *F. spadix* | *P. custodis* |
| *nad2* | ATT | ATT | ATT | T | TAA | TAA |
| *cox1* | ATC | ATC | ATC | TAA | TAA | TAA |
| *cox2* | ATG | ATG | ATG | TAA | TAA | TAA |
| *atp8* | ATA | ATA | ATT | TAA | TAA | TAA |
| *atp6* | ATG | ATG | ATG | TAA | TAA | TAA |
| *cox3* | ATG | ATG | ATG | TAA | TAA | TAA |
| *nad3* | ATA | ATT | ATC | TAG | TAG | TAG |
| *nad5* | ATA | ATG | ATG | TAA | TAA | T |
| *nad4* | ATG | ATG | ATG | T | TA | T |
| *nad4L* | ATG | ATG | ATG | TAA | TAA | TAA |
| *nad6* | ATT | ATA | ATT | TAA | TAA | TAA |
| *cytb* | ATG | ATG | ATG | TAA | TAA | TAG |
| *nad1* | ATG | ATG | ATT | TAA | TAA | TAA |

**TABLE S6.** tRNA site analyses of 3 species fleas of the subfamily Amphipsyllinae.

| tRNA | alignment length | Number of conserved sites | Conserved site ratio (%) | Number of mutation sites | Proportion of mutant ratio (%) |
| --- | --- | --- | --- | --- | --- |
| *trnC* | 67 | 53 | 79.1 | 14 | 20.9 |
| *trnA* | 65 | 61 | 93.8 | 4 | 6.2 |
| *trnD* | 68 | 57 | 83.8 | 11 | 16.2 |
| *trnE* | 67 | 60 | 89.5 | 7 | 10.5 |
| *trnF* | 73 | 60 | 82.2 | 13 | 17.8 |
| *trnG* | 63 | 60 | 95.2 | 3 | 4.8 |
| *trnH* | 65 | 52 | 80.0 | 13 | 20.0 |
| *trnI* | 63 | 61 | 96.8 | 2 | 3.2 |
| *trnK* | 70 | 65 | 92.8 | 5 | 6.2 |
| *trnL1* | 62 | 60 | 96.7 | 2 | 3.3 |
| *trnL2* | 64 | 57 | 89.0 | 7 | 11.0 |
| *trnM* | 68 | 61 | 89.7 | 7 | 10.3 |
| *trnN* | 65 | 62 | 95.4 | 3 | 4.6 |
| *trnP* | 64 | 61 | 95.3 | 3 | 4.7 |
| *trnQ* | 69 | 68 | 98.5 | 1 | 1.5 |
| *trnR* | 64 | 52 | 81.2 | 12 | 18.8 |
| *trnS1* | 69 | 66 | 95.6 | 3 | 4.4 |
| *trnS2* | 66 | 61 | 92.4 | 5 | 7.6 |
| *trnT* | 68 | 56 | 82.3 | 12 | 17.7 |
| *trnW* | 67 | 60 | 89.5 | 7 | 10.5 |
| *trnY* | 63 | 58 | 92.0 | 5 | 8.0 |
| *trnV* | 68 | 61 | 89.7 | 7 | 10.3 |

**TABLE S7.** Divergence times for nodes/clades in the order Siphonaptera. All estimates are represented in millions of years ago (Mya), and “&” represents the relationship of two branches.

| Nodes/Clades | Mean Divergence time (Mya) | 95 % HPD Range (Mya) |
| --- | --- | --- |
| Boreidae & (((Ceratophyllidae + Leptopsyllidae) + ((Vermipsyllidae + Hystrichopsyllidae) + Ctenophthalmidae)) + (Pulicidae + Pygiopsyllidae)) | 119.30 | 105.49-132.61 |
| (Pulicidae + Pygiopsyllidae) & ((Ceratophyllidae + Leptopsyllidae) + ((Vermipsyllidae + Hystrichopsyllidae) + Ctenophthalmidae)) | 101.21 | 90.73-117.42 |
| Pygiopsyllidae & Pulicidae | 95.36 | 82.79-106.98 |
| (Ceratophyllidae + Leptopsyllidae) & ((Vermipsyllidae + Hystrichopsyllidae) + Ctenophthalmidae) | 86.26 | 75.01-99.98 |
| *Frontopsylla diqingensis* & *Frontopsylla spadix* | 40.32 | 34.66-45.53 |


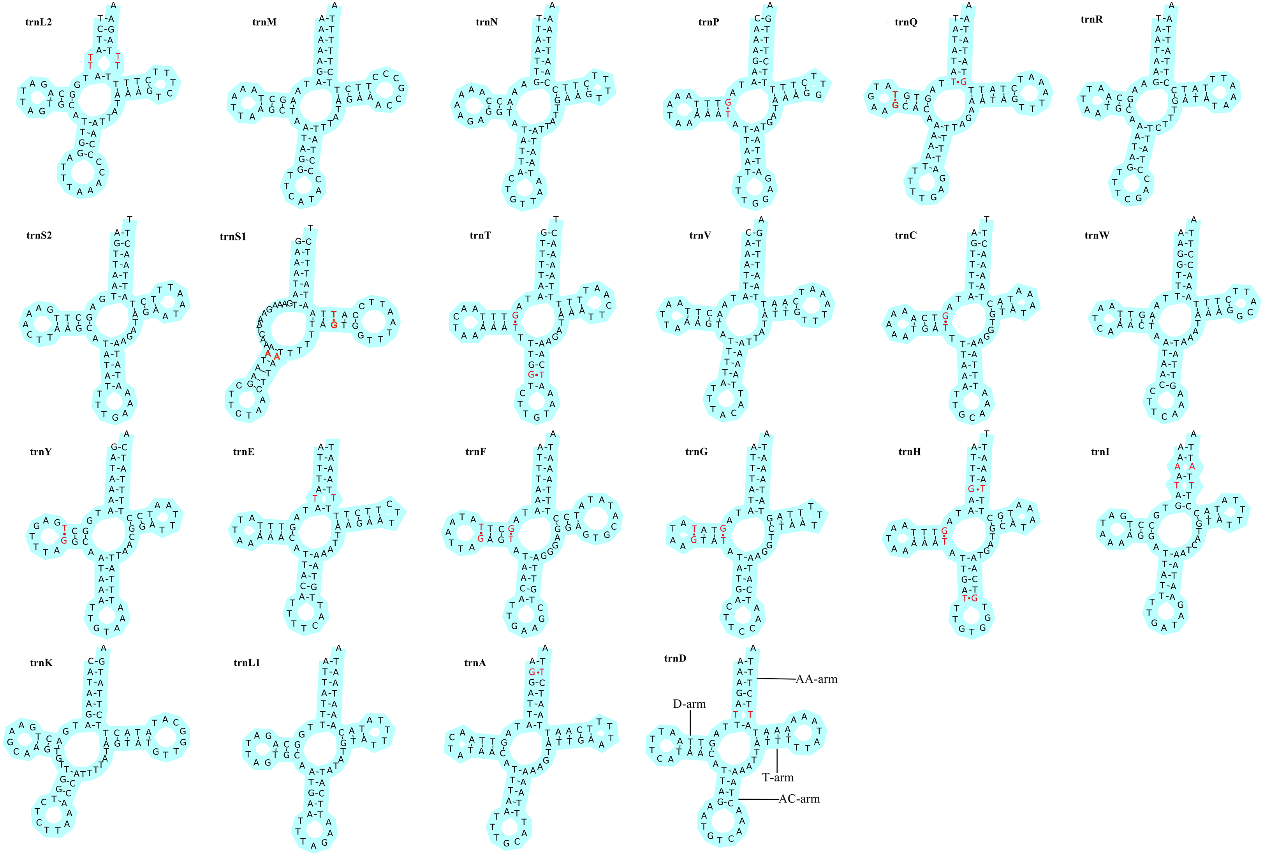


**FIGURE S1.** Predicted secondary structure of 22 tRNAs of *F. diqingensis.* Mismatch base pairs indicated in red.

**
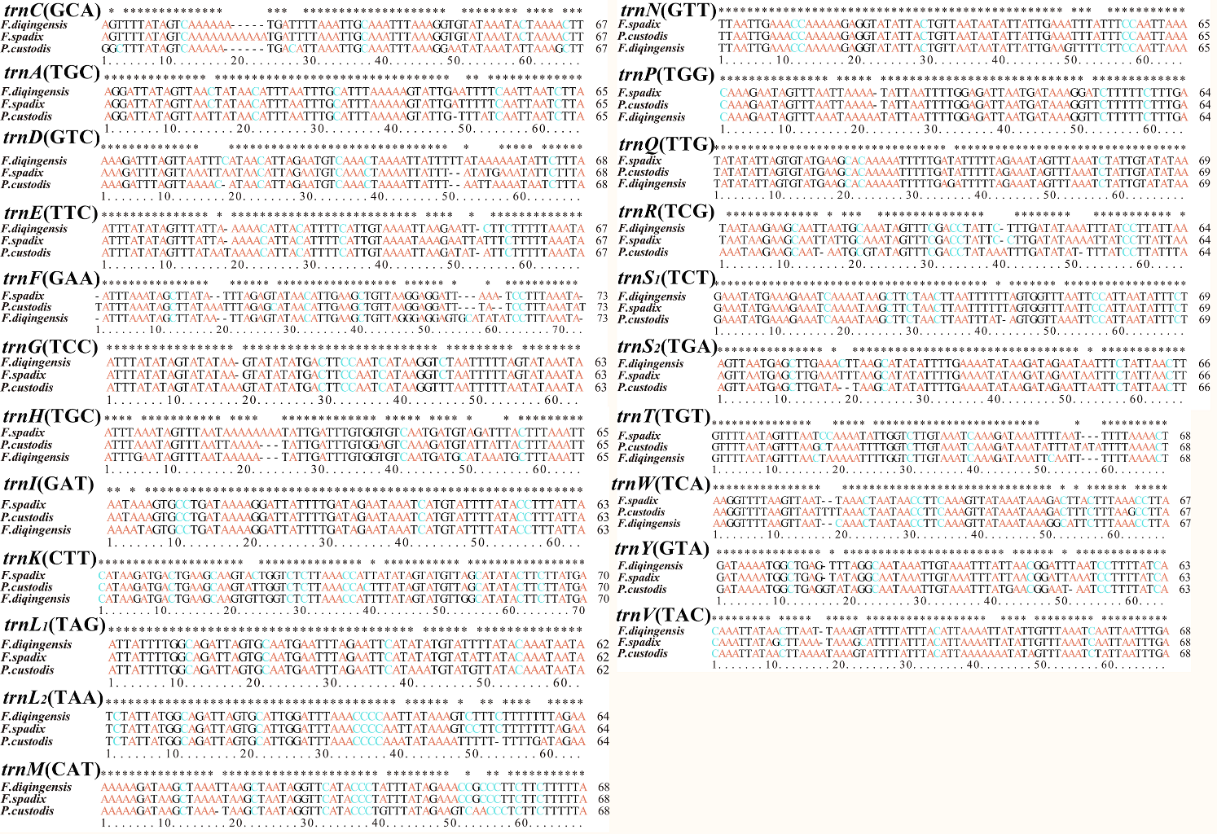
**

**FIGURE S2.** tRNA alignment of 3 species of the subfamily Amphipsyllinae


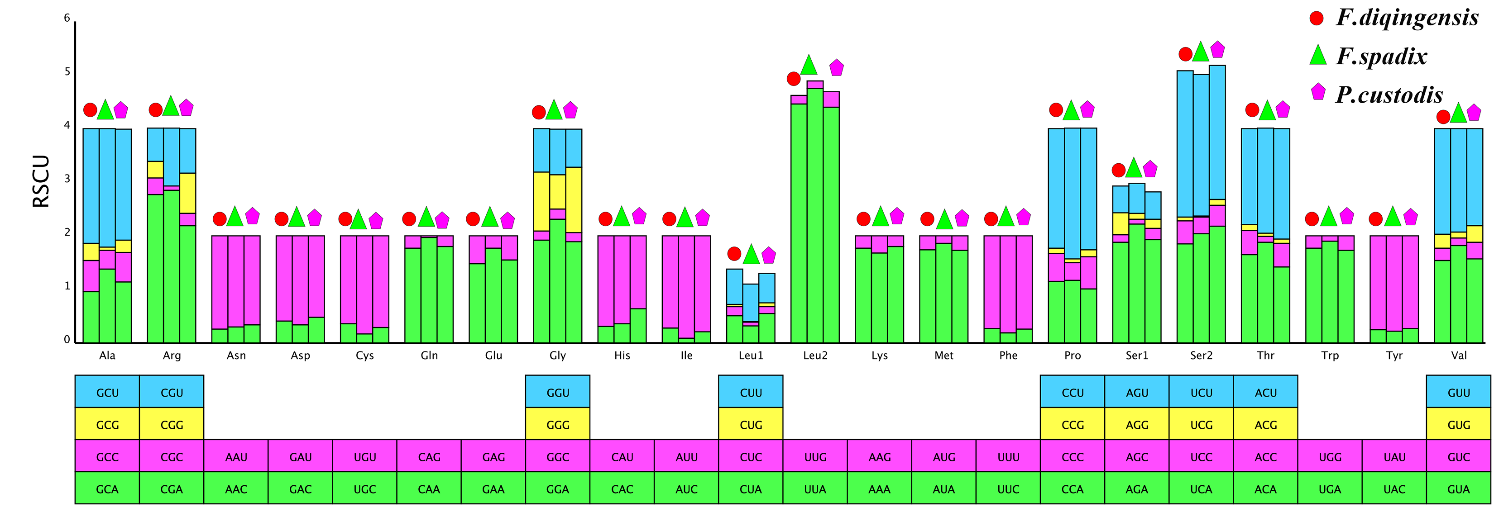


**FIGURE S3.** Relative synonymous codon usage (RSCU) of the subfamily Amphipsyllinae. The Y-axis represents the RSCU value, and the X-axis represents the codons corresponding to each amino acid.


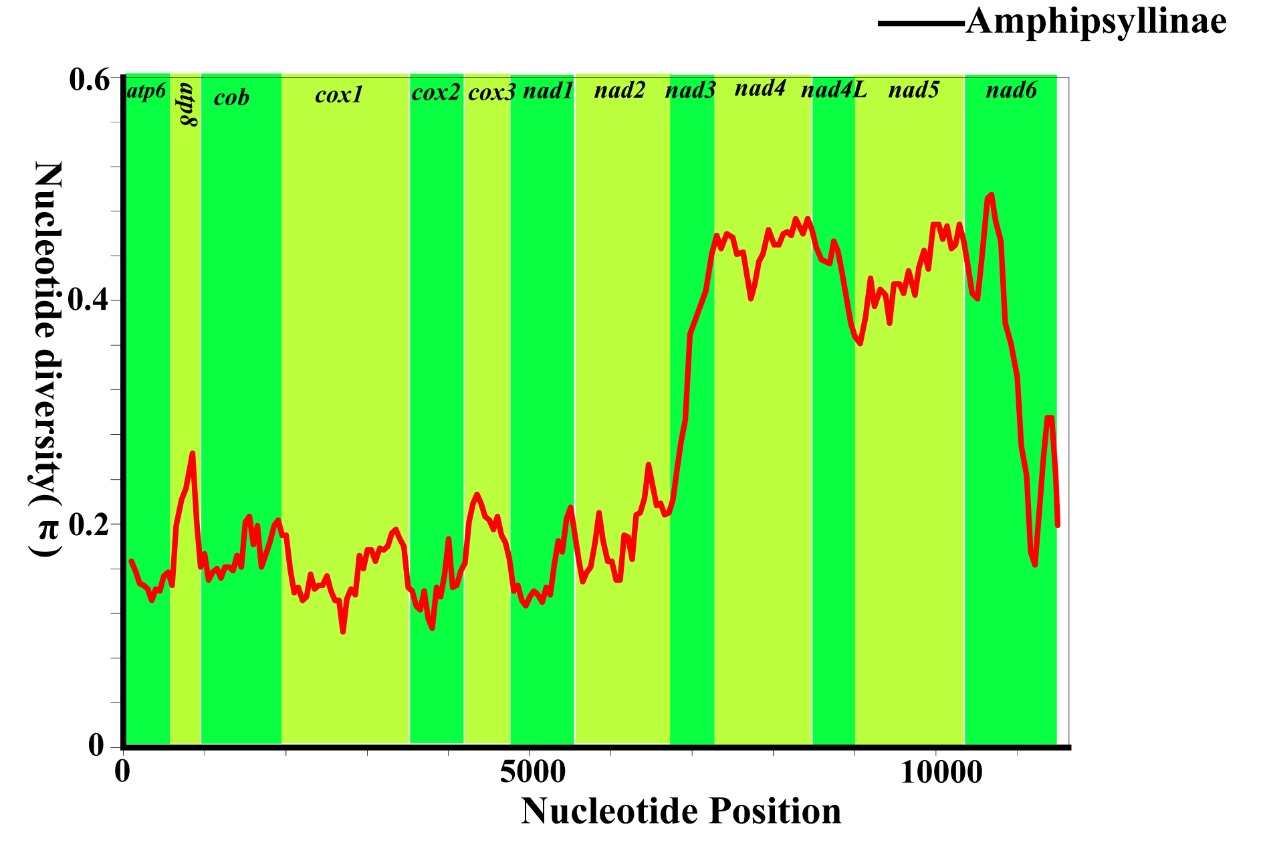


**FIGURE S4.** Analysis of nucleotide diversity in the subfamily Amphipsyllinae.
